# Effectiveness of Therapies Based on Mirror Neuron System to Treat Gait in Patients with Parkinson’s Disease—A Systematic Review

**DOI:** 10.3390/jcm11144236

**Published:** 2022-07-21

**Authors:** Silvia Lahuerta-Martín, Rocío Llamas-Ramos, Inés Llamas-Ramos

**Affiliations:** 1 Physiotherapy Area, Department of Surgery, Ophthalmology, ORL and Physiotherapy, Faculty of Health Sciences, Universidad de Valladolid, 42004 Soria, Spain; silvia.lahuerta@uva.es; 2 Rehabilitation Centre “El Carmen”, C/García Galdeano, 3, 50004 Zaragoza, Spain; 3 Department of Nursing and Physiotherapy, Universidad de Salamanca, C/Donantes de Sangre s/n, 37007 Salamanca, Spain; inesllamas@usal.es; 4 University Hospital of Salamanca, Paseo de San Vicente, 182, 37007 Salamanca, Spain

**Keywords:** Parkinson’s disease, mirror neurons, gait, action observation, motor imagery

## Abstract

Parkinson’s disease (PD) is a neurodegenerative disease that alters gait patterns from early stages. The visuo-motor training strategies such as action observation (AO) and motor imagery (MI) that are based on the activity of the mirror neuron system (MNS) facilitate motor re-learning. The main purpose of this systematic review was to analyze the current scientific evidence about the effectiveness of MNS’s treatments (AO and MI) to treat gait in patients with PD. Searches were completed from the databases PubMed, Web of Science, and PEDro between November and December 2021. The following keywords were used: “Parkinson disease”, “mirror neurons”, “gait”, “action observation”, and “motor imagery”. Randomized control trials of the last 5 years written in English or Spanish were included. Two independent reviewers screened the articles and applied the eligibility criteria, and a third reviewer assisted in this process. A total of six articles were included for final revision. The risk of bias was assessed with the PEDro Scale. The effects of AO and MI using different outcome measures were referenced in terms of disease severity, quality of life, balance, and gait. Training with AO and MI are effective in improving disease severity, quality of life, balance, and gait in patients with PD.

## 1. Introduction

Parkinson’s disease (PD) is a progressive neurodegenerative disease characterized by the loss of dopaminergic neuronal cells located in the substantia nigra and the presence of protein inclusions called Lewy bodies, which include insoluble alpha-synuclein aggregates [1,2,3]. In addition, other regions of the central nervous system (CNS) such as the locus coeruleus and non-dopaminergic neurons may be affected. Its incidence increases with age; it has an onset at 70 years-old, being more prevalent in men than in women in all age groups [4,5,6].

PD is characterized by a range of clinical manifestations that can be divided into non-motor and motor. Non-motor symptoms of PD include depression, hyposmia, sleep disorders, depression, constipation, and sensory disturbances [7,8,9]. The presence of an increased sensory threshold and impaired sensorimotor integration influences the perception of these patients, leading to difficulties in the execution of appropriate motor patterns and a dependence on visual cues for reaching and grasping movements or the gait cycle [7,10]. In more advanced stages, cognitive deficits and alterations of the autonomic nervous system (ANS) are common [1,2,3,11]. The main motor symptoms are resting tremor, rigidity, and bradykinesia. These symptoms lead to a decrease in postural control, affecting balance and gait. However, stability may be affected but not gait and vice versa [12,13].

Gait is affected from the early stages of the disease and its worsening runs parallel to the progression of the pathology where three phases could be established [14]:

- Early phase: decrease in speed and stride length, being less automatic.
- Intermediate phase: greater alteration of gait spatiotemporal parameters. Gait festination becomes evident and freezing (“brief episodes of absence or marked reduction of forward progression of the feet, even though there is an intention to walk”) appears [15,16]. Such freezing involves difficulty in initiating walking onset, walking in confined spaces, or walking with time limitation. All of them are related to an increased risk of falls.
- Advanced stage: increase in blockages and the appearance of dyskinesias. Technical aids such as canes or wheelchairs are used to counteract the increased risk of falls.

Treatment of gait are focused on medication, brain surgery, and physiotherapy [12,16]. The more used medication is levodopa, and regarding brain surgery, deep brain stimulation (DBS) in the subthalamic nuclei is the most used option. Both treatments lead to an improvement in spatiotemporal parameters of gait and freezing of gait (FOG), obtaining good results from the beginning up to two years after the intervention, but becoming less evident with the progression of PD [12,16,17]. Finally, physiotherapy as a complement to medication and DBS is essential. The benefits obtained are based on the use of exercise as a driver of neuronal plasticity [18]. Exercise is related to an increase in synaptic strength, as well as the synthesis of neurotransmitters and neurotrophic factors [19]. This results in improved global functional connectivity of the nervous system.

It is important to consider the cognitive performance level of the patient, as his or her learning from practice will be influenced by feedback, attentional demands, and motivation to perform the task [18,20,21]. In this sense, the motor plan elaborated in the motor cortex can be accessed through the memory circuits.

It is worth highlighting the existing evidence that shows how therapy based on external sensory signals (auditory and visual) [22,23,24] and visuo-motor training help in the process of ideation and motor planning [12,15,18,20,21], emphasizing their participation in FOG improvement.

Action observation (AO) and motor imagery (MI) are two of the most important visuo-motor training strategies, being based on the activation of the mirror neuron system (MNS) to facilitate motor learning [18].

The MNS is a specialized group of neurons located in parietofrontal and limbic systems [25]. The parietofrontal MNS involves premotor cortex, parietal lobe, and the caudal part of the inferior frontal gyrus. The limbic system involves the insula and the anterior mesial frontal cortex. Other structures have been described like supplementary motor area, cerebellum, and primary and secondary somatosensory cortex. The MNS is characterized by being excited when the individual performs an action and when he observes another individual performing or imagining himself performing the action. Several studies have determined that the MNS is made up of visuomotor, audiovisual, and sensory neurons, which are activated when performing the action and through vision, hearing, and proprioception [25,26,27,28,29,30].

The characteristics of the MNS make it possible to establish its role in the understanding and intentionality of the actions of other individuals by comparing them with one’s own experience [26,27,28,31]. As a result of these cognitive processes, motor planning and motor learning or relearning take place [32].

The usefulness of the MNS in physiotherapy treatment lies in the fact that its activity precedes the non-mirror neurons. This makes it possible to predict both the goal of the action and the possible sequence of steps to reach that goal. Non-mirror neurons are only activated during the execution of the action [31].

Recent studies have been able to establish the relationship between the application of AO and MI techniques, with an improvement in the PD patient’s clinical condition in terms of motor relearning [33,34,35]. 

AO therapy is based on the observation of videos in which actions performed by an individual are presented and observed by the patient. This viewing is usually followed by the execution of the same action, although this last step may not be performed [36]. The person in the video performing the action may be the patient himself or a third party. The videos are usually recorded from different perspectives [32,37,38,39] and the most appropriate application protocol has not been established [36], although applications do not exceed 30 min.

Motor imagery (MI) is based on the patient imagining himself performing an action, preferably in the first person (in the absence of muscular movement) and subsequently executing this action [40,41,42,43]. This technique favors motor learning through the activation of the MNS, and there is scientific evidence that shows that when the time of imagination and execution of the action is similar, neuroplasticity is favored [44]. Furthermore, during the execution of the technique, ANS signs can be observed, such as an increase in respiratory rate or heart rate, which are related to the magnitude of the imagined effort [45]. As for AO therapy, MI has not got a main application protocol, although long applications deal with mental fatigue [33,46].

AO and MI have important advantages: they are non-invasive, safe, low-cost therapies and they can be performed at home [33].

Considering the existing literature on MNS-based approaches and their application to the treatment of gait in neurodegenerative diseases such as PD, a systematic review of the most current literature is proposed. This will provide scientific evidence regarding possible changes in the spatiotemporal parameters of gait, the balance, the disease severity, and the quality of life of PD patients.

## 2. Materials and Methods

### 2.1. Search Question

The research question has been elaborated following the PICO format (patient, intervention, control, outcome). This systematic review aims to answer the following question:

In the PD patient, is gait treatment using MNS-based approaches of greater benefit than conventional treatment in terms of spatiotemporal gait parameters, balance, disease severity, and quality of life?

### 2.2. Search Strategy

This Systematic Review follows the recommendations of the PRISMA 2020 Declaration [47]. The protocol for this review has been pre-registered in PROSPERO under ID 298471.

The search and selection of the studies included in the review was conducted by two independent researchers (L.M.-S. and L.R.-R.), to comply with the peer review criteria. In the case of a discrepancy, a third reviewer (L.R.-I.) decided to achieve a consensus. The following electronic databases were used: PubMed, Web of Science, and PEDro. The search strategy was limited from 2017 to November 2021 in all databases (Appendix A).

The inclusion criteria were as follows: articles with samples composed by individuals with idiopathic PD in both the experimental group (EG) and control group (CG); randomized clinical trials (RCTs); articles that used AO/MI as treatments; articles that measured changes in gait parameters; and articles published in English or Spanish. Duplicate articles, articles without access to the full text, articles not describing study results, or studies where AO and MI are used as a means of patient assessment but not treatment were excluded from this review. The following search terms are used in different combinations: “Parkinson’s disease”, “mirror neurons”, “gait”, “action observation”, and “motor imagery” (Annex 1).

### 2.3. Study Selection

The Mendeley ^®^ software was used to manage the bibliography in an orderly fashion.

After contrasting and eliminating duplicate articles, the remaining articles were screened by title and abstract. After screening, a final selection was made on the basis of the eligibility criteria outlined above. This selection was made by two independent reviewers. The final articles that meet these criteria were chosen by both reviewers through discussion and the support of a third reviewer to make a consensus.

### 2.4. Risk of Bias Assessment

The PEDro Scale was used to assess the risk of bias. It is a scale composed of 11 items. This tool is used to assess the methodological quality of RCTs by evaluating their credibility or internal validity (items 2–9), and whether the study contains sufficient statistical information that can be interpreted (items 10 and 11). Item 1 assesses external validity and is therefore not included in the final score. For each completed item, a score of 1 is given, with a maximum of 10.

The use of the PEDro scale is supported by scientific evidence, indicating its reliability and validity [48].

## 3. Results

**Figure 1:**
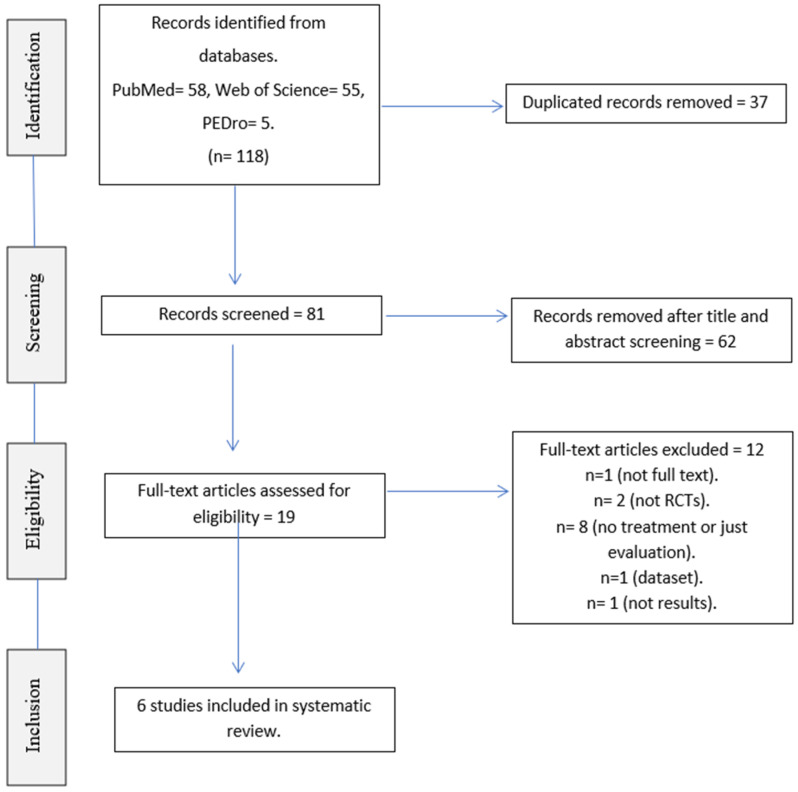
PRISMA flowchart.

After database searching, 118 articles were potentially selected. Duplicates were eliminated [37], and manuscripts were read by title and abstract; 62 articles were removed because they did not meet the inclusion criteria. At a second time, 19 articles were full text reviewed by two researchers independently. In total, six studies met the selection criteria for inclusion in the systematic review (Figure 1).

The selected sample is composed by RCTs evaluating the application of AO treatment (four RCTs) or MI (one RCT), and the AO and MI combination (one RCT) in different intervention protocols. All the studies had EGs and CGs in which the patients had a diagnosis of idiopathic PD. The studies include a total number of 156 patients, as the studies of Mezzarobba et al. [49,50] were conducted with the same sample of patients. Sample sizes varied from 20 to 64 patients. The mean age of the participants ranged from 63 to 75 years. In all studies, the number of male participants exceeded the number of female participants. The mean number of years with disease was greater than 6 in all studies, except for the study of Agosta et al. [51], where this was not specified, but which had an inclusion criteria that implies that participants had a diagnosis of disease ≥5 years. Regarding the levodopa daily dose (LEDD) of the participants, it should be noted that most of the studies [49,50,51,52,53] detailed that the amount should be stable from the last 4 [51,52,53] or 8 weeks [49,50]. In four of the studies [49,50,51,54], patients needed to present FOG to be included, while in the remaining studies [52,53], this characteristic was not stated as an inclusion criterion, although gait parameters were evaluated. The data on sociodemographic variables and the main clinical variables to be considered are detailed in Table 1.

All studies detail that assessments were carried out before starting treatment, at the end of treatment, and at the various follow-up periods established. Patients were assessed at the functional level in the ON condition. In addition, all studies showed that treatment in both the EG and CG was also carried out in the ON condition.

The main characteristics of the interventions conducted in each study are detailed in Table 2. The duration of the intervention varied between 2 weeks [52] and 8 weeks [49,50], with a minimum total of 10 sessions [52] and a maximum of 18 sessions [53]. Frequency ranged from 2 to 5 days per week, with 2 days per week being the most common [49,50,54]. Sessions lasted between 45 min [54] and 2 h [52], with the most common period being 60 min [49,50,51,53]. Follow-up after the end of treatment was performed in all studies [49,50,51,53,54], except for the study of Abraham et al. [52]. Follow-ups ranged from 4 [49,51,52,54] weeks to a maximum of 12 weeks [49,50]. Treatments were performed individually in all studies [49,50,51,53], except for that of Abraham et al. [52] and Pelosin et al. [54], which were performed in groups. In addition, all treatments were implemented in a physiotherapy room, except for the CG in the study of Abraham et al. [52], which was performed at home with follow-up by the physiotherapist.

### 3.1. Assessment of Risk of Bias

Once the selection of studies was made, their methodological quality was assessed using the PEDro scale. Table 3 shows the results after completion.

The scores showed three studies with good methodological quality [49,51,53], two studies with fair methodological quality [52,54], and one study with poor methodological quality [50].

### 3.2. Results Analysis

The aim of all the studies in the review [49,50,51,52,53,54] was to determine whether treatment with AO or MI in the different modalities proposed in the EG, compared to other interventions that do not include these MNS-based approaches, led to an improvement in disease severity, cognitive functions, motor functions, quality of life, and activation of different areas of the cerebral cortex. Effectiveness was studied in the short [52] and long term [49,50,51,53,54]. There were no significant differences between the EG and CG in all the studies before the treatments started.

The results of the review in relation to the variables that have been proposed for analysis are shown below.

- Disease severity.

Disease severity improved significantly (*p* < 0.05) with the interventions under study, both if a comparison was made between EG and CG, as well as if EGs were observed in isolation. Moreover, these improvements were maintained during follow-ups [49,51,52,53]. Furthermore, MI produces a significant increase in participation and autonomy, directly influencing the disease severity [52].

- Quality of life.

Quality of life improved significantly after the treatment period in the EG and CG, but it was only maintained at follow-up in the EG [49,51,53]. Comparisons between the two groups did not generally show significant results in favor of the EG [51,53].

AO + sonification showed the most promising results related to quality of life [49].

- Balance.

In general, balance improved after application of the various interventions in EGs and CGs; however, these improvements only remained significant at follow-up in EGs [49,51,54]. Comparison between groups showed mixed results, and therefore it cannot be generalized that AO and MI approaches significantly improve balance [49,50,51,52].

Group treatment of AO [54] and the combination of dual task + AO − MI [53] showed significant results in favor of EGs at the end of treatment and at follow-ups.

- Gait.

Gait improved significantly after interventions in EGs and CGs but was only maintained over time in EGs [49,50,51,53,54]. In the comparison between groups, significant results were obtained in favor of the EGs both at the end of treatment and at different follow-ups [49,50,52,53,54].

It should be noted that FOG obtained significant improvements both at the end of the treatments and at the respective follow-ups of the studies in which it was assessed [49,50,51,53,54].

## 4. Discussion

This systematic review was conducted with the aim of providing the most current evidence on the effectiveness of MNS-based interventions in improving gait in patients with PD, as well as enhancements in disease severity, balance, and quality of life. From our knowledge, this is the first review that specifically addresses walking, with samples of PD patients in the EG and CG [33,36].

After assessing the methodological quality of the studies using the PEDro scale, two studies were of fair quality [52,54] and one of poor quality [50]. The review by Ryan et al. [36] also included the study by Pelosin et al. [54], concluding, through the Cochrane Risk of Bias (RoB) 2.0 tool, whose methodological quality is good. Regarding the study by Mezzarobba et al. [50], its low methodological quality according to the PEDro scale was because it refers to the original article to explain the methodology [49]. Considering that the methodological quality of the original article was 8/10, this result can be extrapolated to the low-scoring article [50]. Therefore, it would be interesting to use several tools to assess the risk of bias of the review articles.

According to Ryan et al. [36], there is level 1 evidence to support AO intervention for the improvement of balance, FOGt, disease severity, and other motor and non-motor skills (aerobic capacity). However, the studies included in this review [36] do not provide the same level of evidence for the improvement of spatiotemporal gait parameters in PD patients. Taking this into account, it would be interesting to prioritize the performance of RCTs of high methodological quality that would allow good evidence to be obtained and strong recommendations to be made.

This review does not include studies assessing the long-term effects of MI in isolation. In a previous review, studies evaluating the long-term effects of MI in PD patients were found [33]. Braun et al. [55] concluded that the combination of MI with physiotherapy did not produce significant improvements in any of the study variables (including gait) compared to the CG in which a combination of physiotherapy and relaxation was applied. Therefore, it would be interesting to follow this line of work to corroborate these results or to add new evidence to the existing one.

The analysis carried out in this review shows promising results in all the variables under study, but there are also certain controversies. The significant improvement in the variables to be analyzed in the EG (disease severity, quality of life, balance, and gait) is evident in all the studies in which they were evaluated with different assessment scales [49,50,51,52,53,54]. When analyzing the EG and CG of the studies separately, significant results were obtained in both for the variables under study at the end of treatment. However, these significant results were maintained at follow-up only in the EG, which leads us to believe that all included interventions based on the MNS allow for long-term improvements in disease severity, quality of life, balance, and gait.

Analyzing the comparison between the EG and CG of the studies in the review, the results were significant in favor of the EG for the variables of disease severity, quality of life, and gait in all the studies. Balance was the only variable that showed differences between studies, so it cannot be generalized that AO and MI interventions lead to a significant improvement in balance compared to conventional physiotherapy treatment in the group comparison. The studies that obtained significant improvements in EG in the group comparison were those of Pelosin et al. [54] and Sarasso et al. [53]. It would be interesting to pursue both lines of research; on the one hand, to explore the effectiveness of MI in group interventions, and on the other hand, interventions could be focused on the use of dual tasks in combination with MI and AO in group interventions.

Regarding the results obtained on FOG, all the studies in which it is evaluated (FOG-Q, NFOG-Q) achieved significant improvements in long-term EG scores [49,51,53,54]. These significant improvements only occurred in group comparisons in the study by Mezzarobba et al. [49]. On the other side, it was demonstrated that PD patients with cognitive function impairment have more risks to develop neuropsychiatric symptoms, depression, and anxiety compared with healthy controls. For that reason, motor symptoms could be a predictor of FOG [56].

Three studies [49,50,51] used auditory cues during AO. Because the use of cues improves kinematics and FOG, sonification is presented as an alternative to visual cues, which generate more dependence, as well as standard auditory cues (metronome), which are less effective in regulating patient coordination [49]. The process of sonification is based on the transformation of kinematic data, of a movement visible in a video, into sounds. This could add additional information of the movement to the patient, so that they could reproduce it in a better spatio-temporal way. A research proposal would be to perform a combined MI protocol with sonification or even AO − MI + sonification.

Sarasso et al. [53] conducted the first study on the effects of joint application of AO and MI in long-term treatment and follow-up in patients with PD. The use of both tools together enhances their effect on the activation of the MNS (motor learning benefits) [35]. In addition, the implementation with a double task (motor-cognitive) allowed for the improvement of cognitive functions such as attention and working memory (which are usually affected in PD from early stages) [57,58]. This evidence opens the door to new intervention protocols that would be interesting to explore.

Two studies in the review [51,53] reported changes in the cerebral cortex function of the EG patients after intervention, with increased activity in areas of the CNS (right inferior frontal gyrus, cerebellum). These findings are related to improvements in disease severity, balance, and gait.

### Limitations

Firstly, the small sample sizes (the maximum is 64 patients) of the studies selected could be a bias, so it would be interesting to conduct RCTs with larger sample sizes. Furthermore, the assessment of methodological quality that has been made by a single scale may be advisable to use at least two scales to compare the results provided by both. Finally, there is a lack of an assessment of the certainty of the evidence and the strength of the recommendations. This assessment would allow the findings obtained in the review to be integrated into clinical practice with sufficient confidence [47].

## 5. Conclusions

Intervention with dynamic neurocognitive imagery improves disease severity, balance, and gait after treatment. Although the AO intervention is effective, its application together with sonification improves disease severity, balance, spatiotemporal gait parameters, and freezing, in the short and long term. Group intervention with AO improves balance, spatiotemporal gait parameters (speed), and freezing, in the short and long term, with the combination of AO − MI + dual task being the most effective. Studies are needed to evaluate the results of intervention with MI for gait improvement in PD in the long term.

## Figures and Tables

**Table 1:** Socio-demographic and clinical variables (baseline).

		Agosta et al. [51]	Abraham et al. [52]	Mezzarobba et al. [49]	Pelosin et al. [54]	Mezzarobba et al. [50]	Sarasso et al. [53]
Sample size	EG	12	10	12	33	12	13
	CG	13	10	10	31	10	12
Age	EG	69.0 ± 8.0	66.4 ± 12.5	74.67 ± 5.93	70.4 ± 4.5	74.67± 5.93	67.51 ± 6.12
	CG	64.0 ± 7.0	65.1 ± 7.5	72 ± 5.87	72.8 ± 3.1	72 ± 5.87	63.81 ± 9.23
Gender	EG	10 M/2 W	9 M/1 W	7 M/5 W	16 M/17 W	7 M/5 W	8 M/5 W
	CG	8 M/5 W	7 M/3 W	7 M/3 W	15 M/16 W	7 M/3 W	8 M/4 W
Treatment type		AO	DNI	AO + Sonification	AO	AO + Sonification	Dual task + AO − MI
Years of illness	EG	≥5	6.1 ± 3.8	10.75 ± 3.44	10.7 ± 3.9	10.75 ± 3.44	8.08 ± 4.13
	CG	≥5	8.5 ± 4.5	9.4 ± 4.86	9.5 ± 4.2	9.4 ± 4.86	7.92 ± 3.53
LEDD (mg)	EG	897 ± 508	NA	972.5 ± 253.17	435.2 ± 158.5	972.5 ± 253.17	757.23 ± 480.49
	CG	988 ± 345	NA	983.22 ± 379.58	383.1 ± 270.2	983.22 ± 379.58	555 ± 217.25
FOG		YES	YES	YES	YES	YES	YES
UPDRS III	EG	27.6 ± 9.7	38.4 ± 13.8	32.92 ± 8.69	31.6 ± 6.1	32.92 ± 8.69	26.27 ± 9.88
	CG	23.5 ± 7.9	32.1 ± 12.2	33.2 ± 13.99	30.9 ± 7.2	33.2 ± 13.99	28.83 ± 8.47
Hoehn and Yahr Scale (mean ± SD)	EG	2.3 ± 0.4	2.0 (1.8, 2.5) *	2.33 ± 0.49	2.4 ± 0.5	2.33 ± 0.49	2.3
	CG	2.2 ± 0.3	2.0 (2.0, 2.5) *	2.3 ± 0.67	2.6 ± 0.3	2.3 ± 0.67	2.4
PDQ-39	EG	24.7 ± 11.1	NA	51.67 ± 26.9	NA	51.67 ± 26.9	18.19 ± 7.52
	CG	20.2 ± 11.6	NA	50.8 ± 29.43	NA	50.8 ± 29.43	18.95 ± 12.16

Clinical variables measured before the start of treatment and ON status. * Median (first and third quartiles). EG: experimental group. CG: control group. M: men. W: women. AO: action observation. DNI: dynamic neuro-cognitive imagery. MI: motor imagery. LEDD: levodopa equivalent daily dose. Mg: milligrams. FOG: freezing of gait. UPDRS III: Unified Parkinson’s Disease Rating Scale III. SD: standard deviation. PDQ-39: 39-item Parkinson’s Disease Questionnaire. NA: not available.

**Table 2:** Study characteristics.

Study	Sample and Treatment	Duration	Measurements	Results
Agosta et al. [51]	N = 25 EG (12): **Observation**: 2 videos presenting strategies to avoid FOG episodes with auditory signal associated. Each video lasts 6 min and was repeated twice. Six videos in total. **Execution**: 8 min after each viewing. Accompanied by auditory signals. CG (13): **Observation**: static landscape sequences videos. **Execution**: same actions as EG under physiotherapist instructions.	60 min/day. 3 sessions/week. 4 weeks.	PRE, POST, and 4 weeks follow-up. (i) Disease severity: HY, UPDRS III. (ii) Freezing severity: FOG-Q, UPDRS II. (iii) Motor function: 10 MWT, BBS. (iv) Quality of life: PDQ-39. (v) fMRI.	EG: POST: *p* < 0.05 in FOG-Q, UPDRS II ON, UPDRS III ON, PDQ-39, BBS, and 10 MWT. **Week 8**: *p* < 0.05 in UPDRS III ON, PDQ-39, BBS, and 10 MWT. CG: POST: *p* < 0.05 in FOG-Q, PDQ-39, and 10 M WT. **Not maintained in 4 weeks follow-up**. Groups comparisons *p* < 0.05 in UPDRS III ON in POST.
Abraham et al. [52]	N = 20 EG (10): **DNI**: DNI warm-up (15 min), DNI concept introduction and practice A (35 min), rest (10 min), DNI concept introduction and practice B (35 min), DNI movement session (20 min), and DNI cool-down (5 min). CG (10): **Home intervention**: health-related texts reading (1.5 h) and 30-min exercise video target on PD impairments. Telephone follow-up 3 times over the treatment.	2 h/day. 5 sessions/week. 2 weeks.	PRE and 2–5 days POST intervention. (i) Disease severity: UPDRS, ABC, IPA, BPI, BDI: II. (ii) Motor function: TUG, Fwd gait, 6 MWT, 360° turn test, PRT, 30 s chair stand. (iii) Cognitive function: Trail Making test, BSM, BPST, Reverse Corsi Blocks Visuospatial task. (iv) Imaginary Measures: MIQ-RS, KVIQ-20, VMIQ-2. (v) Satisfaction questionnaire.	EG vs. CG: POST. *p* < 0.05 in all imaginary measures except for MIQ-RS kinesthetic and VMIQ-2 kinesthetic. (i) IPA *p* < 0.05. (ii) *p* < 0.05 in TUG- manual, 360° turn test, PRT. (iii) BPST span *p* < 0.05. EG is more improved than CG.
Mezzarobba et al. [49]	N = 22 EG (12): **Observation + sonification**: 8 audio-video (1.5 min each one) of 8 specific gait motor gestures related to FOG. Each video twice. Execution: repetition of the same actions (1.5 min each action) after each video observation. CG (10): Execution: same 8 gestures using visual and auditory cues to facilitate the learning of spatial-temporal parameters. Both groups were supervised by a physiotherapist.	60 min/day. 2 sessions/week. 8 weeks.	PRE, POST, and 1- and 3-month follow-ups. (i) NFOG-Q (main outcome). (ii) Disease severity: UPDRS, HY. (iii) Quality of life: PDQ-39. (iv) Motor function: MPAS, TUG, 6 MWT, BBS. (v) Neuropsychological evaluation.	**EG significant improvements compared to CG** (*p* < 0.05) in NFOG-Q, PDQ-39 (mobility and discomfort subscales), and UPDRS III. Results are maintained at 3 months follow-up. BBS significant improvements in EG.
Pelosin et al. [54]	N = 64. Group intervention. EG (33): **Observation**: 2 videos/session (from a total of 6) (6 min/video) with strategies for circumventing FOG. **Execution**: practice strategies under supervision. Progressive increase in video complexity. CG (31): Observation: 2 videos/session, with sequences of static pictures, with same duration as EG. Execution: same actions and amount of time than EG.	45 min/day. 2 sessions/week. 5 weeks.	PRE, POST, and 4 weeks follow-up after treatment ends. (i) FOG-Q (ii) TUG (iii) BBS (iv) 10 MWT	**PRE vs. POST**: significant changes (*p* < 0.05) in all variables of EG and CG. **PRE vs. 4 weeks follow-up**: *p* < 0.05 in all variables of EG and in 10 MWT of CG.
Mezzarobba et al. [50]	N = 22 EG (12): Observation + sonification: 8 audio-video/session (1.5 min each one) of 8 specific gait motor gestures, related to FOG. Each video twice. Execution: repetition of the same actions (1.5 min each action) after each video observation. CG (10): Execution: same 8 gestures using visual and auditory cues to facilitate the learning of spatial-temporal parameters. Both groups were supervised by a physiotherapist.	60 min/day. 2 sessions/week. 8 weeks.	PRE, POST, and 1- and 3-months follow-ups. (i) Postural control: sit-to-walk task (STW). Measured: COM and COP in 6 positions task-specific events.	EG: **COM**: no significance. **COP**: significant differences PRE vs. 3 months follow-up in percentiles 10–35 and 63–72. Heel take-off is performed earlier and the STW task is significantly shorter in duration. The COP is significantly lower. CG: COM and COP no significant changes.
Sarasso et al. [53]	N = 25 EG (13): **Dual task + AO − MI**. Four gait/balance exercises each session (2 min observation task + 5 min execution task + 2 min imagination task + 5 min execution task). CG (12): **Dual task**. Four gait/balance exercises combined with video observation of static landscape instead of observation-imagination. Difficulty increases during the treatment of EG and CG to include dual-task.	60 min/day. 3 sessions/week. 6 weeks.	PRE, POST, and 8 weeks follow-up. (i) Disease severity: HY, UPDRS, ABC. (ii) Quality of life: PDQ-39. (iii) Gait: TUG, 10 MWT, NFOG-Q (iv) Balance: MiniBESTest. (v) Cognitive evaluation: AST. (vi) Scanner MRI. Motor task (feet dorsiflexion) with eyes closed. Dual task: mentally count from 100 subtracting by 3.	EG: **POST and 8 weeks follow-up**. *p* < 0.05 in TUG, TUG-COG, TUG-MAN, its relative peak and rotational speed, DTC during TUG-COG, DTC on the average rotational speed of TUG-COG, MiniBESTest, ABC, 10 MWT, NFOG-Q, and PDQ-39. **POST**: *p* < 0.05 in MDS-UPDRS III OFF, HY OFF, and AST. CG: POST: *p* < 0.05 in TUG, TUG-COG, TUG-MAN, DTC in average rotational speed in TUG-COG, MDS-UPDRS III OFF, and AST. Eight weeks **follow-up**: *p* < 0.05 in TUG, TUG-COG, TUG-MAN, MDS-UPDRS III ON, and rotational speed during TUG- COG. EG vs. CG. **POST and 8 weeks follow-up**: EG significative improvements regarding CG in TUG-COG, average and peak rotational speed in TUG-COG, peak rotational speed in TUG, TUG-MAN, DTC during TUG- COG, MiniBESTest, 10 MWT (normal speed), and ABC.

HY: Hoehn and Yahr scale. UPDRS: Unified Parkinson’s Disease Rating Scale. MDS-UPDRS: Movement Disorders Society Unified Parkinson’s Disease Rating Scale. FOG-Q: Freezing of Gait Questionnaire. NFOG- Q: New Freezing of Gait Questionnaire. 10 MWT: 10 Meters Walking Test. BBS: Berg Balance Scale. PDQ-39: Parkinson’s Disease Questionnaire—39 items. Min: minute. DNI: dynamic neurocognitive imagery. ABC: Activities Specific Balance Confidence Scale. IPA: Impact on Participation and Autonomy Scale Questionnaire. BPI: Brief Pain Inventory. BDI-II: Beck Depression Inventory-II. TUG: time up and go test. Fwd gait: forward gait speed. 6 MWT: 6 Minutes Walking Test. PRT: Push and Release Test. BSM: Brooks Spatial Memory Task. BPST: Body Position Spatial Task. MIQ-RS: Movement Imagery Questionnaire—Revised Second Version. KVIQ-20: Kinesthetic and Visual Imagery Questionnaire—20 items. VMIQ-2: Vividness of Movement Imagery Questionnaire—Revised Version. PRE: pre-treatment. POST: post-treatment. MPAS: Modified Parkinson’s Activity Scale. Mini-BESTest: Mini Balance Evaluation Systems Test. TUG-COG: time up and go + cognitive. TUG-MAN: time up and go + manual. DTC: dual task cost. AST: attention switching task.

**Table 3:** Risk of bias: PEDro Scale.

	Agosta et al. [51]	Abraham et al. [52]	Mezzarobba et al. [49]	Pelosin et al. [54]	Mezzarobba et al. [50]	Sarasso et al. [53]
Eligibility criteria	Yes	Yes	Yes	Yes	No	Yes
Random participant allocation	Yes	Yes	Yes	Yes	Yes	Yes
Concealed allocation	Yes	No	Yes	No	No	Yes
Groups similar at baseline	Yes	Yes	Yes	Yes	No	Yes
Subjects blinding	No	No	No	No	No	No
Therapist blinding	No	No	No	No	No	No
Assessor blinding	Yes	No	Yes	No	No	Yes
Less than 15% dropouts	No	No	Yes	Yes	No	Yes
Intention to treat analysis	No	No	Yes	No	No	No
Between groups statistical comparisons	Yes	Yes	Yes	Yes	Yes	Yes
Point measures and variability data	Yes	Yes	Yes	Yes	Yes	Yes
Total	6/10	4/10	8/10	5/10	3/10	7/10

## Data Availability

The datasets generated and/or analyzed during the current study are available from the corresponding author on reasonable request.

## Appendix A. Search Strategy

PubMed:

(Parkinson disease) AND (mirror neurons)

(Parkinson disease) AND (gait) AND (action observation)

(Parkinson disease) AND (gait) AND (motor imagery)

((gait[MeSH Terms] OR gait) AND (parkinson disease[MeSH Terms] OR Parkinson Disease) AND (action observation OR motor imagery)).

((parkinson disease[MeSH Terms]) OR (parkinson disease)) AND ((mirror neurons) OR (mirror neurons[MeSH Terms])).

Web of Science:

(Parkinson disease) AND (gait) AND (action observation)

(Parkinson disease) AND (gait) AND (motor imagery) NOT (action observation)

PEDro:

(Parkinson disease) AND (gait) AND (action observation)

(Parkinson disease) AND (gait) AND (motor imagery)
